# Untargeted Metabolomics Combined with Bioassay Reveals the Change in Critical Bioactive Compounds during the Processing of Qingzhuan Tea

**DOI:** 10.3390/molecules26216718

**Published:** 2021-11-06

**Authors:** Peng-Cheng Zheng, Chun-Yin Qin, Pan-Pan Liu, Lin Feng, Tie-Jun Ling, Jing-Ming Ning, Liang Zhang, Xiao-Chun Wan

**Affiliations:** 1 State Key Laboratory of Tea Plant Biology and Utilization, Anhui Agricultural University, Hefei 230036, China; zpct15@163.com (P.-C.Z.); qin_chunyin@163.com (C.-Y.Q.); zuotianyujintian@163.com (L.F.); lingtj@ahau.edu.cn (T.-J.L.); ningjm@ahau.edu.cn (J.-M.N.); 2 Institute of Fruit and Tea, Hubei Academy of Agricultural Sciences, Wuhan 430064, China; liuppitea@163.com

**Keywords:** qingzhuan tea, metabolomics, pile-fermentation, sensory evaluation, bioactivities

## Abstract

Qingzhuan tea (QZT) is a typical Chinese dark tea that has a long-time manufacturing process. In the present study, liquid chromatography coupled with tandem mass spectrometry was used to study the chemical changes of tea samples during QZT processing. Untargeted metabolomics analysis revealed that the pile-fermentation and turnover (post-fermentation, FT) was the crucial stage in transforming the main compounds of QZT, whose contents of flavan-3-ols and flavonoids glycosides were decreased significantly. The bioactivities, including the antioxidant capacities and inhibitory effects on α-amylase and α-glucosidase, were also reduced after the FT process. It was suggested that although the QZT sensory properties improved following pile-fermentation and aging, the bioactivities remained restrained. Correlation analysis indicated that the main galloylated catechins and flavonoid glycosides were highly related to their antioxidant capacity and inhibitory effects on α-amylase and α-glucosidase.

## 1. Introduction

Qingzhuan tea (QZT), one of the most famous dark teas in China, has attracted significant attention due to its use of special manufacturing technology and long-time pile-fermentation [1,2,3]. The processing steps of QZT include fixing, rolling, preliminary drying to the water content below 13%, pile-fermentation (turnover thrice and aging 3 months), steaming, shaping, and drying [4]. Unlike other dark teas, pile-fermentation sustains QZT from several months to years [5]. After preliminary drying, raw dark teas are piled together up to 3–5 m in a warehouse. The chemical changes of raw dark tea are thought to occur during the long-term pile-fermentation, responsible for the unique flavor and health benefits [6].

To date, the main chemical analysis for QZT has consisted of flavan-3-ols, purine alkaloids, and some low-molecular-weight phenolic acids as the main compounds [7]. Given that the QZT raw materials consist of mature leaves and stems, the chemical composition differs from that of other typical green teas, such as Longjing tea, Maofeng tea, and some black teas [8,9]. Liquid chromatography-tandem mass spectrometry and metabolomics are widely used as a potent tool in tea research, despite differences in the secondary metabolites or the chemical constituents during processing [10]. For example, it was recently uncovered that the first stage of pile-fermentation has the strongest effect on the formation of *N*-ethyl-2-pyrrolidone-substituted flavan-3-ols [11].

Diabetes, a common noncommunicable chronic disease, is one of the causes of morbidity and mortality, and a global health issue [12]. Several studies have shown that tea possesses various health benefits, such as hypoglycemic, lipid-lowering, antioxidant, and anti-inflammatory benefits [13,14,15]. Among the six types of tea, dark tea has been reported to possess the ability of regulating gut microbiota, thereby mediating anti-obesity effects [16]. However, there are limited studies on the relationship between the bioactivities of compounds and the processing of dark tea. Therefore, it is necessary to study the relationship to optimize the processing technology. 

Compared with Pu-erh tea and Fu brick tea [5], which belong to the same category of dark tea, reports of the chemical changes of QZT during processing are limited. In our previous study, we preliminarily studied the changes of the main compounds during pile-fermentation [17] and found that the polyphenols drastically decreased at the beginning of pile-fermentation [18,19]. However, the changes of main compounds and trace metabolites during the whole processing steps were less studied. In the present study, we aimed to reveal the comprehensive changes of main and trace metabolites of QZT, and the relationship of the tea compounds and biological activities. 

## 2. Materials and Methods

### 2.1. Samples and Chemical Reagents

To make the QZT, fresh tea leaves (FTL) were collected from the Zhaoliqiao tea factory (Chibi, China) and then fixed for 3 min at 280 °C (collected sample was labeled as DTL). Then, the tea leaves were rolled for 15 min (collected sample was labeled as RTL) by a rolling machine (6CR-55, Zhejiang, Shang Yang Co., Ltd., Quzhou, China), and finally dried in the sun (collected sample was labeled as CT). Then, CT underwent pile-fermentation after adding 30% water, and the tea pile was turned over three times during the pile-fermentation with an interval of 7–10 days. All tea samples (after turning over) were collected and labeled as 1st FT (the first pile-fermented tea), 2nd FT (the second pile-fermented tea), 3rd FT (the third pile-fermented tea), respectively. Whereafter, the pile-fermented tea was aged for 6 months at room temperature (25 °C), and the aged tea leaves were collected at an interval of 3 months and labeled as 1MAT (1-month aged tea), 3MAT (3-month aged tea), 6MAT (6-month aged tea), respectively. Finally, the QZT was obtained after drying and labeled as DT (dried tea). All collected samples from the different stage of QZT processing were freeze-dried using a lyophilizer (Christ Alpha 1-4 LSC basic, Lower Saxony, Germany) and stored at −80 °C prior to analysis.

Gallic acid (GA, >98%), caffeine (CAF, >98%), theobromine (THB, >98%), (+)—catechin (C, >98%), (−)—epicatechin (EC, >98%), (-)—gallocatechin (GC, >98%), (−)—epigallocatechin (EGC, >98%), (−)—gallocatechin gallate (GCG, >98%), (−)—epigallocatechin gallate (EGCG, >98%), and (−)—epicatechin gallate (ECG, >98%) were purchased from Yuanye Biotechnology Company (Shanghai, China). LC-MS-grade acetonitrile, methanol, and water were purchased from Thermo Fisher Scientific Co. (Fair Lawn, NJ, USA). Other reagents were of analytical grade.

### 2.2. Sample Preparation

The dried tea sample (100 mg) was extracted with 4 mL of 70% methanol (*v*/*v*) under ultrasonic extraction for 10 min, and then centrifuged for 10 min at 6000 rpm. The tea sample was extracted again. The supernatants were combined and transferred into a 10 mL of the flask, and then diluted to 10 mL with methanol. The content was filtered via a 0.22 µm nylon membrane prior to high-performance liquid chromatography (HPLC) and liquid chromatography coupled with quadrupole time of flight mass spectrometry (LC-Q-TOF-MS) analysis.

### 2.3. Sensory Evaluation

The color, odor, and taste of tea infusion and the brewed tea leaves were evaluated according to a standardized methodology for tea analysis (GB/T 23776-2018). In brief, 5.0 g of tea were brewed with 250 mL of boiled water for 5 min. Then, the tea infusion was evaluated by panelists who were trained for 4 weeks.

### 2.4. Determination the Contents of Main Compounds by HPLC

The HPLC system (Agilent Technologies, Palo Alto, CA, USA) which consisted of an Infinity binary pump, integrated vacuum degasser, autosampler, thermostated column compartment, and diode array detector (DAD), was used to determine the contents of tea polyphenols and purine alkaloids. The HPLC (Waters 2695, Milford, PA, USA) was coupled with a Waters Phenomenex C18 (250 × 4.6 mm, 5 µm) column, UV detector (detection wavelength set at 280 nm), and a 2998 PDA detector. The temperature of the column was kept constant at 40 °C. The mobile phase was composed of 2% (*v*/*v*) acetic acid (A) and acetonitrile (B). The gradient elution was set as follows: 0 min, 93.5% A; 16 min, 15% A; 16–25 min, 25% A; 30 min, 93.5% A; and 40 min, 93.5% A. The injection volume was 10 µL and the flow rate was 1.0 mL/min. The analytical method was based on our previously published method [20].

### 2.5. Untargeted Metabolomics Analysis

The metabolites of QZT samples were analyzed using an Agilent 1290 LC system (Agilent Technologies, Palo Alto, CA, USA) coupled to a time-of-flight mass spectrometer (Agilent Technologies, Palo Alto, CA, USA). The separation of the chemical compounds was the same as our established method [13]. Samples analyses were performed in triplicate. The mass spectrometer was operated in the negative ionization mode at a resolving power of 40,000 over a full scan range of *m*/*z* 100–1200 with the following settings: sheath gas temperature, 350 °C; nebulizer, 35 psi; gas flow, 8 L/min; gas temperature, 320 °C; sheath gas flow, 11 L/min.

SMICA-P 13.0 software (Umetrics, Umeå, Sweden) was used for multivariate analysis based the LC-MS data. Data within a 95% confidence interval were accepted.

### 2.6. Inhibitory Effects of QZT Samples on α-Amylase and α-Glucosidase

The QZT samples from different processing stages were prepared similarly to the method described in Section 2.2. Then, the tea infusions were diluted to various concentrations for the determination of their inhibition effects on α-amylase and α-glucosidase. The in vitro inhibition of α-amylase and α-glucosidase of each tea sample was assessed using a previously described methodology, and results were expressed as the inhibition rate [20].

### 2.7. In Vitro Antioxidant Assay

The in vitro antioxidant activity of each tea sample was evaluated by the following three tests. The ferric reducing ability of plasma (FRAP) was determined according to the method proposed by Benzie and Strain (1997), and the results were expressed as the FRAP value [21]. The antioxidant activity in relation to the 2, 2-diphenyl-1-picrylhydrazyl (DPPH) radical was determined by the method proposed by Brand-Williams et al. (1995), and the results were expressed as the DPPH scavenging rate and IC_50_ value [22]. For the ABTS assay, the antioxidant activity of each sample was determined according to the published method [23].

### 2.8. Statistical Analysis

All samples were analyzed in triplicate, and the results were expressed as mean ± SD (n = 3). One-way ANOVA followed by Tukey’s test was used to separate the means. *p*-values below 5% were considered significant.

## 3. Results and Discussion

### 3.1. Sensory Evaluation

**Figure 1:**
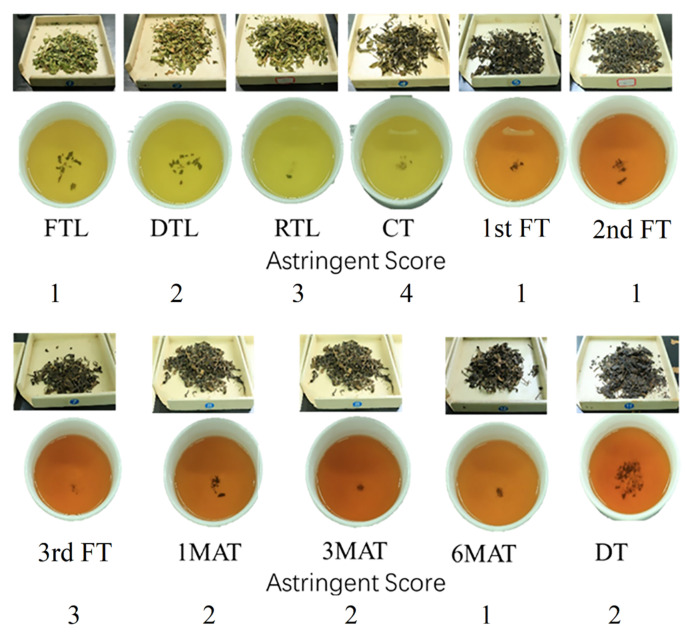
Sensory evaluation on the color and taste (mainly astringency) of QZT samples from the different processing stages. The first line above the abbreviations of tea samples showed the color of tea leaves, and the second line showed the color of tea infusions. The numbers under the abbreviations of tea samples represent their corresponding astringent scores.

As shown in Figure 1, with the depth of QZT processing, the color of tea infusion and tea leaves gradually turned red and darker. Furthermore, the astringent scores of the processed tea samples showed a significant variation during processing. This suggested that tea leaves underwent dramatic changes in chemical compositions during the QZT processing. Initially, the astringent score was gradually increased along with the processing steps, but after pile-fermentation and aging of crude tea, the astringent score was dramatically decreased. Hence, the flavor of QZT may be mainly formed at the pile-fermentation and aging steps, especially the third FT and 1MAT. The enzymatic oxidation of tea polyphenols has been inhibited or terminated due to the polyphenol oxidase was deactivated during the fixation of fresh tea leaves, whereas the oxidation of tea polyphenols and other processing-derived products might play an important role in the color and taste formation of QZT products [24].

### 3.2. Contents of Main Compounds in QZT Samples

The contents of the main compounds in the QZT samples from different processing steps are presented in Table 1. The aqueous extract is an important factor in evaluating the quality of tea samples [25]. With the processing of QZT, the content of aqueous extracts (%) did not show significant changes, but it was statistically declined. Furthermore, the contents of total polyphenols, amino acids, flavonoids, and soluble sugars were continuously decreased along with processing. These results are consistent with most of the similar reports for Chinese dark teas, such as Pu-erh tea and Fuzhuan tea [25,26,27].

Gallic acid was gradually accumulated during the QZT processing. Specially, after the third pile-fermentation, its content (%) attained the highest value at 0.46% (Table 1). However, similar to the other reports of Chinese dark tea [28], the content of caffeine was relatively stable. In the beginning, the fresh tea leaves contained 2.80% of caffeine, but after whole processing, the caffeine content remained at 2.47%.

### 3.3. Contents of Catechins during Processing

As shown in Table 2, the content of each catechin compound was determined. EGCG was the main polyphenol in the fresh tea leaves, which was continuously decreased with processing. The crude tea leaves also had a higher content of EGCG. However, after three times of pile-fermentation, its content was significantly decreased. Other galloylated catechins showed a similar variation, and the contents of non-galloylated catechins, including GC, EGC, C, and EC, were also decreased. These results suggest the hydrolysis of galloylated catechins may not produce non-galloylated catechins as the final product. The non-galloylated catechins were also decreased, and then the total catechins were greatly reduced. Subsequently, the astringent perception was correspondingly changed.

### 3.4. Multivariate Analysis of LC-MS Based Metabolomics Data

**Figure 2:**
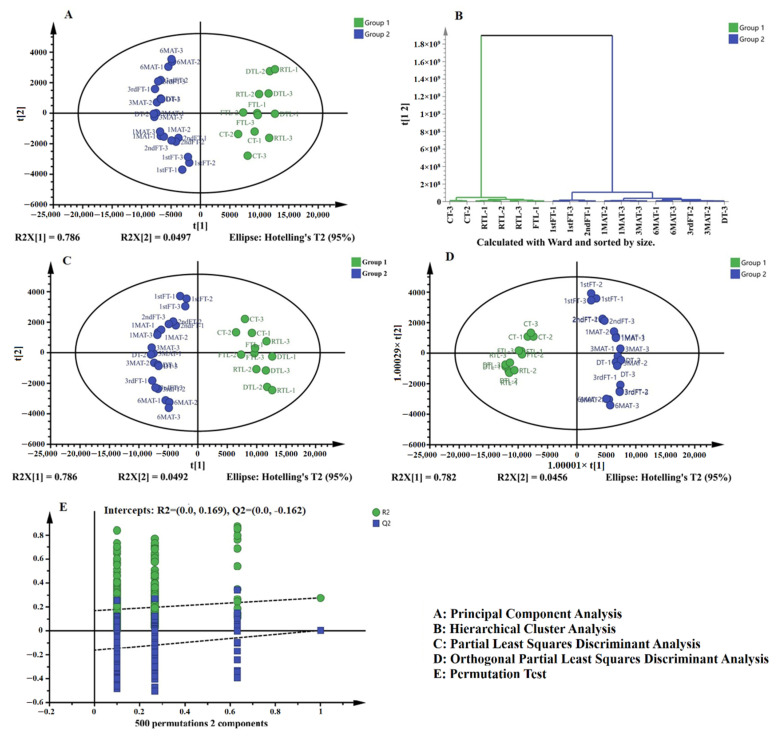
Multivariate analysis based on the LC-Q-TOF-MS data of QZT samples from different processing stages.

All of MS data files were uploaded to the MS-DIAL (version 2.74) for data processing, including the retention time (min), *m*/*z* value, and intensity alignment. The processed dataset was analyzed by SMICA-P 13.0 multivariate statistical software. The unsupervised principal composition analysis (PCA), supervised partial least squares discriminant analysis (PLS-DA), and orthonormal partial least squares discriminant analysis (OPLS-DA) discriminated all tested tea samples into two categories (Figure 2). One cluster contained FTL, DTL, RTL, and CT, while the other tea samples (1st FT, 2nd FT, 3rd FT, 1MAT, 3MAT, 6MAT, and DT) were grouped in the same cluster. The hierarchical cluster analysis (HCA) also distinguished all samples in the same way. These results indicated that the first four processing steps, including FTL, DTL, RTL, and CT, which were also the main steps for processing green tea, had similar chemical constituents. Once the tea sample underwent pile-fermentation, the chemical constitutes changed.

**Figure 3:**
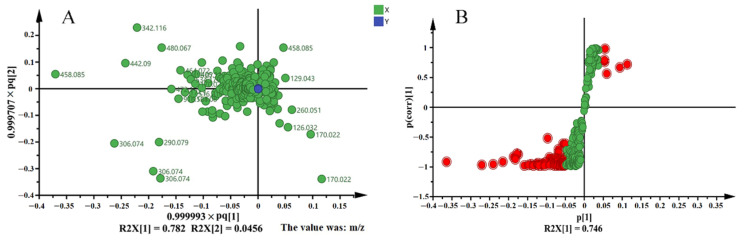
Metabolomics analysis of QZT samples from different processing stages. (**A**) Scatterplots based on OPLS-DA analysis between the two categories of QZT samples. (**B**) *S*-plots based on OPLS-DA analysis between the two categories of QZT samples.

To further explore the marker compounds responsible for the variation between these two categories of tea samples, the scatterplots and *S*-plot were profiled, as shown in Figure 3. All compounds having VIP >1 were considered as important compounds (marker compounds) responsible for the classification of QZT samples. In total, 41 marker compounds were tentatively identified in Table 3 by referencing the chemical standards and mass fragments. These compounds mainly belonged to the polyphenols, which were susceptible to the pile-fermentation process. Among these compounds, galloylated catechins primarily contributed to the changes of chemical constitutes, such as EGCG, EGC, C, and ECG. Furthermore, some astringent compounds were also identified as marker compounds, which suggested the content decreasing of these compounds may attribute to the astringent score decreasing of tea samples, for instance, flavonoid glycosides and procyanidins.

Based on the above results, it was obvious that first pile-fermentation was the critical processing step for the transformation of chemical constituents in QZT. Our previous studies only concerned the chemical changes of pile-fermentation, while the first and second pile-fermentation were also recognized as important processing steps [17]. Thus, in the present study, we comprehensively studied the chemical compounds in QZT samples from various processing stages (in total, 11 processed tea samples were collected and analyzed) and found that significantly more marker compounds were affected by QZT processing. To correlate the relationship between chemistry and biological activities, all processed samples were studied with respect to their antioxidant capacities and the inhibitory effects on α-amylase and α-glucosidase. The contribution of each compound to biological activity was also calculated by the correlation coefficient.

### 3.5. Inhibitory Effects on α-Amylase and α-Glucosidase

**Figure 4:**
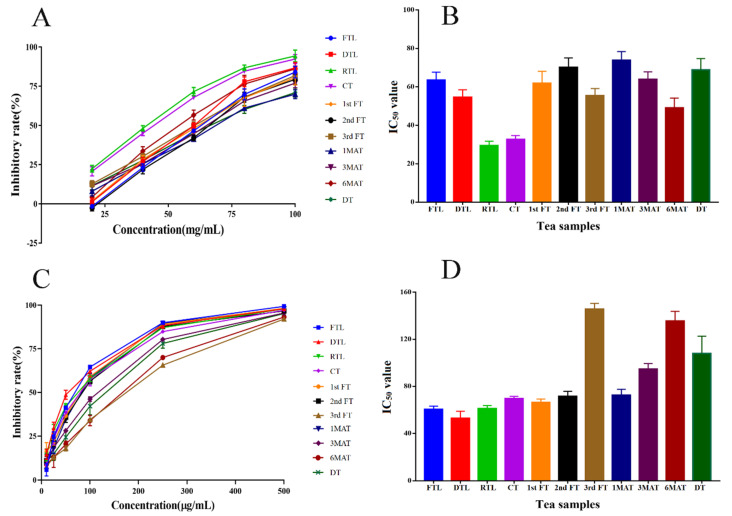
The inhibitory effects of different QZT samples on α-amylase and α-glucosidase. (**A**) Inhibitory rates of different QZT samples with various concentrations on α-amylase. (**B**) IC_50_ values of different QZT samples on α-amylase: FTL: 63.48 mg/mL; DTL: 54.59 mg/mL; RTL: 29.47 mg/mL; CT: 32.59 mg/mL; 1st FT: 61.93 mg/mL; 2nd FT: 70.16 mg/mL; 3rd FT: 55.42 mg/mL; 1MAT: 73.86 mg/mL; 3MAT: 63.98 mg/mL; 6MAT: 49.07 mg/mL; DT: 68.88 mg/mL. (**C**) Inhibitory rates of different QZT samples with various concentrations on α-glucosidase. (**D**) IC_50_ values of different QZT samples on α-glucosidase: FTL: 60.54 μg/mL; DTL: 52.91 μg/mL; RTL: 61.01 μg/mL; CT: 69.57 μg/mL; 1st FT: 66.26 μg/mL; 2nd FT: 71.60 μg/mL; 3rd FT: 145.60 μg/mL; 1MAT: 72.54 μg/mL; 3MAT: 94.72 μg/mL; 6MAT: 135.57 μg/mL; DT: 107.94 μg/mL.

In Figure 4, an apparent regulation could be observed, as the RTL and CT samples showed the best inhibition effects on both α-amylase and α-glucosidase. The two samples belonged to the unfermented tea samples, because since the first FT, the crude tea started pile-fermentation for a different time. This suggested that the original compounds of QZT before pile-fermentation showed stronger inhibition effects on α-amylase and α-glucosidase, with lower IC_50_ values at 29.47–63.48 mg/mL (α-amylase) and 52.91–69.57 µg/mL (α-glucosidase). Correspondingly, in Table 2, the contents of the main catechins were significantly decreased after the first fermentation and turnover (1st FT). The non-targeted metabolomics results also confirmed that the first four samples were classified into the same category.

### 3.6. Antioxidant Activities

**Figure 5:**
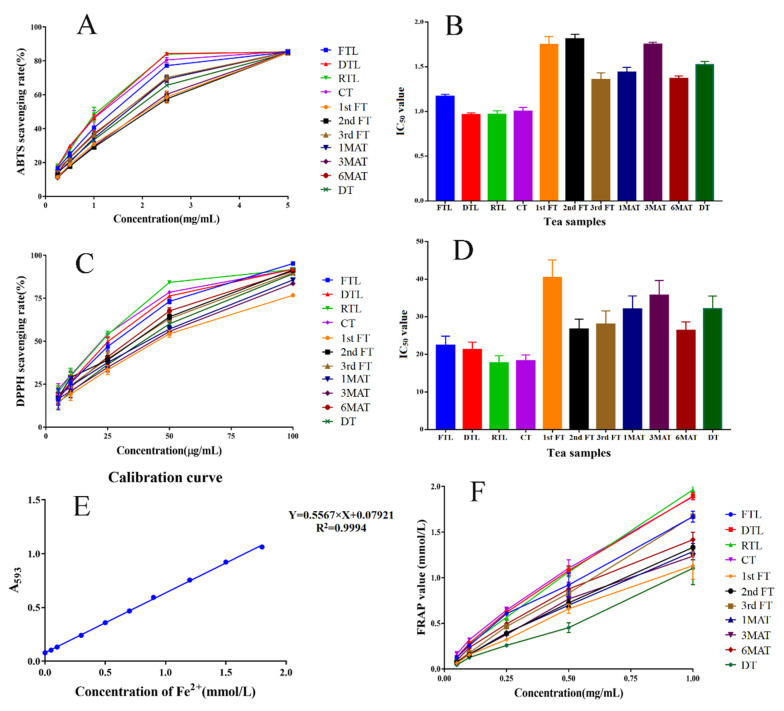
The antioxidant activity of different samples during QZT processing. (**A**) ABTS scavenging rates of different QZT samples. (**B**) IC_50_ values of different QZT samples on ABTS scavenging: FTL: 1.17 mg/mL; DTL: 0.96 mg/mL; RTL: 0.97 mg/mL; CT: 1.00 mg/mL; 1st FT: 1.75 mg/mL; 2nd FT: 1.81 mg/mL; 3rd FT: 1.36 mg/mL; 1MAT: 1.44 mg/mL; 3MAT: 1.75 mg/mL; 6MAT: 1.37 mg/mL; DT: 1.52 mg/mL. (**C**) DPPH scavenging rates of different QZT samples. (**D**) IC_50_ values of different QZT samples on DPPH scavenging: FTL: 22.38 μg/mL; DTL: 21.27 μg/mL; RTL: 17.72 μg/mL; CT: 18.28 μg/mL; 1st FT: 40.43 μg/mL; 2nd FT: 26.69 μg/mL; 3rd FT: 27.99 μg/mL; 1MAT: 32.03 μg/mL; 3MAT: 35.71 μg/mL; 6MAT: 26.38 μg/mL; DT: 32.14 μg/mL. (**E**) The calibration curve of Fe^2+^. (**F**) FRAP values of different QZT samples with different concentrations (0.05–1 mg/mL).

Figure 5 demonstrates the effect of different tea samples on antioxidant activity and the radical scavenging ability. We observed similar results to the inhibition effects on α-amylase and α-glucosidase. The samples before pile-fermentation and turnover (before the 1st FT) had stronger antioxidant activities compared with the pile-fermented and aged samples. This suggested that a lower content of total phenolic compounds in tea samples were strongly correlated to their antioxidant activity.

### 3.7. Correlation Analysis between Chemical Compositions and Biological Activities

From the above results about chemical analysis and biological activities, including the antioxidant and inhibition effects on carbohydrate hydrolases, it can be seen the chemical compositions and biological activities of QZT samples were both varied during the processing. Polyphenols are usually considered the main bioactive compounds of tea [29]. In the present study, we also conducted a correlation analysis between metabolites and bioactivities. As shown in Table 4, the total polyphenols and flavonoids were positively correlated to the antioxidant capacity and inhibitory effects on α-amylase and α-glucosidase. These results suggested the polyphenols were the main bioactive compounds of tea. Among all the polyphenols, the Pearson correlation coefficients of EGCG, GCG, ECG, EGC, and GC were greater than 0.5. Other polyphenols, including quercetin-3-*O*-glucopyranosyl-*O*-glucopyranosyl-*O*-rhamnoside, myricetin-3-*O*-galactoside, and methoxy-epigallocatechin gallate, were also strongly related to tea’s biological activities.

## 4. Conclusions

Pile-fermentation had stronger effects on chemical compositions and biological activities, including antioxidant capacities and inhibitory effects on α-amylase and α-glucosidase. QZT usually needs long-term processing, especially for pile-fermentation and aging, whereas the biological activities of tea samples were reduced during the processing with the decreasing of polyphenols. Galloylated catechins and flavonoids glycosides were also degraded after pile-fermentation, which is similar to the results of ripened Pu-erh tea during post-fermentation. However, the loss of catechins and flavonol glycosides, which mainly belong to the astringent and bitter compounds, will contribute to the improvement of sensory quality of QZT and hence increase the consumer acceptance. The present study indicated that the flavor (especially the astringent taste) of the mature tea leaves was markedly improved after long-term pile-fermentation and aging, whereas its biological activities were correspondingly decreased. Therefore, the fermentation and aging processing time of QZT need be controlled and optimized.

## Figures and Tables

**Table 1:** The contents of aqueous extracts, total polyphenols, total amino acids, total flavonoids, soluble sugar, gallic acid, caffeine, and theobromine of different processed tea samples (% *wt/wt*).

Samples	Aqueous Extracts	Total Polyphenols	Total Amino Acids	Total Flavonoids	Total Soluble Sugars	Gallic Acid	Caffeine	Theobromine
FTL	37.71 ± 2.21 ^a^	18.18 ± 1.06 ^a^	1.37 ± 0.08 ^a^	2.24 ± 0.03 ^a^	5.92 ± 0.62 ^a^	0.08 ± 0.00 ^c^	2.80 ± 0.16 ^a,b^	0.08 ± 0.00 ^d^
DTL	35.57 ± 0.51 ^a,b^	16.33 ± 1.11 ^a,b^	1.13 ± 0.04 ^a,b^	2.07 ± 0.02 ^b^	5.49 ± 1.18 ^a^	0.09 ± 0.00 ^c^	2.57 ± 0.09 ^c,d^	0.07 ± 0.00 ^e^
RTL	36.31 ± 0.63 ^a,b^	15.99 ± 0.85 ^b^	0.95 ± 0.04 ^b^	2.09 ± 0.02 ^b^	5.69 ± 0.49 ^a^	0.07 ± 0.01 ^c^	2.50 ± 0.11 ^c,d^	0.07 ± 0.00 ^f^
CT	35.47 ± 0.52 ^a,b^	15.61 ± 0.49 ^b^	0.83 ± 0.25 ^b,c^	1.95 ± 0.09 ^b^	4.93 ± 0.05 ^a,b^	0.15 ± 0.01 ^bc^	2.92 ± 0.15 ^a^	0.09 ± 0.00 ^b,c^
1st FT	34.69 ± 0.15 ^b^	12.87 ± 0.34 ^c^	0.76 ± 0.12 ^b,c^	1.78 ± 0.11 ^c^	6.05 ± 0.39 ^a,b^	0.07 ± 0.00 ^c^	2.59 ± 0.09 ^c^	0.09 ± 0.00 ^c^
2nd FT	35.14 ± 0.29 ^b,c^	9.77 ± 0.97 ^d^	0.69 ± 0.06 ^b,c^	1.63 ± 0.05 ^d^	4.79 ± 0.46 ^a,b^	0.08 ± 0.01 ^c^	2.70 ± 0.13 ^b^	0.10 ± 0.00 ^b^
3rd FT	34.70 ± 0.33 ^b,c^	7.08 ± 0.23 ^e^	0.57 ± 0.04 ^c^	1.55 ± 0.03 ^d,e^	3.99 ± 0.52 ^a,b^	0.46 ± 0.04 ^a^	2.81 ± 0.10 ^b^	0.08 ± 0.00 ^e^
1MAT	34.16 ± 0.38 ^b,c^	5.58 ± 0.24 ^e,f^	0.53 ± 0.02 ^c,d^	1.51 ± 0.02 ^d,e^	3.26 ± 0.42 ^b^	0.43 ± 0.04 ^a^	2.65 ± 0.15 ^b,c^	0.09 ± 0.00 ^c,d^
3MAT	34.03 ± 0.52 ^b,c^	5.14 ± 0.11 ^f^	0.51 ± 0.03 ^c,d^	1.47 ± 0.02 ^e^	3.32 ± 0.53 ^b^	0.43 ± 0.02 ^a^	2.54 ± 0.14 ^c^	0.06 ± 0.00 ^f^
6MAT	34.19 ± 0.43 ^b,c^	4.52 ± 0.31 ^f^	0.47 ± 0.01 ^d^	1.43 ± 0.02 ^e^	3.33 ± 0.30 ^b^	0.37 ± 0.01 ^a^	2.38 ± 0.13 ^d^	0.08 ± 0.00 ^e^
DT	32.72 ± 0.91 ^c^	4.16 ± 0.17 ^f^	0.43 ± 0.01 ^d^	1.44 ± 0.03 ^e^	3.25 ± 0.21 ^b^	0.25 ± 0.02 ^b^	2.47 ± 0.13 ^c,d^	0.11 ± 0.01 ^a^

Note: The abbreviations for samples are FTL: fresh tea leaves; DTL: deactivated tea leaves; RTL: rolled tea leaves; CT: crude tea; 1st FT: the first pile-fermented tea; 2nd FT: the second pile-fermented tea; 3rd FT: the third pile-fermented tea; 1MAT: 1-month aged tea; 3MAT: 3-month aged tea; 6MAT: 6-month aged tea; and DT: dried tea. Different letters in the same column indicated that the contents of chemical compounds had significant differences (*p* < 0.05).

**Table 2:** The contents of catechins in QZT samples from different processing stages (% *wt/wt*).

Samples	GC	EGC	C	EGCG	EC	GCG	ECG	CG	Total Catechins	Galloylated/Non-Galloylated Catechins
FTL	0.98 ± 0.02 ^a^	1.36 ± 0.04 ^a^	0.22 ± 0.01 ^b^	3.28 ± 0.09 ^a^	0.62 ± 0.03 ^a,b^	0.84 ± 0.04 ^b^	0.94 ± 0.04 ^a^	0.17 ± 0.01 ^b,c^	8.41 ± 0.17 ^a^	1.64 ± 0.02 ^c,d^
DTL	0.91 ± 0.01 ^b^	1.18 ± 0.05 ^b^	0.25 ± 0.00 ^a^	3.03 ± 0.10 ^a,b^	0.54 ± 0.01 ^b,c^	1.01 ± 0.07 ^a^	0.79 ± 0.02 ^b^	0.20 ± 0.01 ^a,b^	7.91 ± 0.19 ^a,b^	1.74 ± 0.02 ^b^
RTL	0.64 ± 0.03 ^c^	1.37 ± 0.04 ^a^	0.19 ± 0.01 ^c^	3.08 ± 0.08 ^a,b^	0.50 ± 0.01 ^c^	0.81 ± 0.04 ^b^	0.83 ± 0.03 ^b^	0.16 ± 0.01 ^b,c^	7.58 ± 0.14 ^b^	1.80 ± 0.03 ^a^
CT	0.65 ± 0.01 ^c^	1.22 ± 0.03 ^b^	0.18 ± 0.01 ^c^	2.85 ± 0.08 ^b^	0.67 ± 0.03 ^a^	0.58 ± 0.03 ^c^	0.82 ± 0.03 ^b^	0.12 ± 0.01 ^c^	7.09 ± 0.15 ^b,c^	1.60 ± 0.03 ^d^
1st FT	0.56 ± 0.02 ^d^	1.00 ± 0.06 ^c^	0.24 ± 0.01 ^a,b^	2.70 ± 0.12 ^bc^	0.57 ± 0.03 ^b^	0.42 ± 0.03 ^d^	0.75 ± 0.02 ^b^	0.23 ± 0.02 ^a^	6.47 ± 0.18 ^c^	1.73 ± 0.02 ^b^
2nd FT	0.60 ± 0.01 ^c,d^	1.00 ± 0.02 ^c^	0.18 ± 0.01 ^c^	2.20 ± 0.06 ^c^	0.43 ± 0.03 ^d^	0.62 ± 0.03 ^c^	0.60 ± 0.02 ^c^	0.11 ± 0.01 ^c,d^	5.74 ± 0.12 ^c,d^	1.60 ± 0.04 ^d,e^
3rd FT	0.40 ± 0.02 ^e^	0.85 ± 0.04 ^d^	0.14 ± 0.01 ^d^	1.62 ± 0.08 ^d^	0.29 ± 0.03 ^e,f^	0.48 ± 0.04 ^d^	0.50 ± 0.01 ^d^	0.17 ± 0.02 ^b^	4.45 ± 0.15 ^d^	1.66 ± 0.01 ^c^
1MAT	0.38 ± 0.01 ^e^	0.63 ± 0.02 ^e^	0.11 ± 0.00 ^f^	1.06 ± 0.03 ^e^	0.23 ± 0.02 ^f^	0.27 ± 0.01 ^e^	0.36 ± 0.02 ^e,f^	0.09 ± 0.01 ^c,d^	3.13 ± 0.08 ^e^	1.32 ± 0.02 ^g^
3MAT	0.30 ± 0.01 ^f^	0.46 ± 0.01 ^f^	0.11 ± 0.01 ^e,f^	0.98 ± 0.03 ^e^	0.32 ± 0.01 ^e^	0.28 ± 0.01 ^e^	0.42 ± 0.02 ^e^	0.08 ± 0.02 ^d^	2.95 ± 0.07 ^e^	1.48 ± 0.03 ^f^
6MAT	0.14 ± 0.01 ^g^	0.27 ± 0.01 ^g^	0.13 ± 0.01 ^d,e^	0.67 ± 0.02 ^f^	0.29 ± 0.02 ^e,f^	0.23 ± 0.01 ^e^	0.32 ± 0.01 ^f^	0.09 ± 0.02 ^c,d^	2.14 ± 0.05 ^f^	1.57 ± 0.04 ^d,e^
DT	0.15 ± 0.03 ^g^	0.23 ± 0.01 ^g^	0.09 ± 0.00 ^f^	0.20 ± 0.02 ^g^	0.24 ± 0.01 ^f^	0.14 ± 0.01 ^f^	0.35 ± 0.02 ^e,f^	0.08 ± 0.01 ^c,d^	1.48 ± 0.04 ^f^	1.09 ± 0.08 ^h^

Note: The abbreviations for samples are FTL: fresh tea leaves; DTL: deactivated tea leaves; RTL: rolled tea leaves; CT: crude tea; 1st FT: the first pile-fermented tea; 2nd FT: the second pile-fermented tea; 3rd FT: the third pile-fermented tea; 1MAT: 1-month aged tea; 3MAT: 3-month aged tea; 6MAT: 6-month aged tea; and DT: dried tea. Different letters in the same column indicated that the contents of chemical compounds had significant differences (*p* < 0.05).

**Table 3:** The critical compounds responsible for the classification of QZT samples from different processing stages.

No.	*t_R_*(min)	MW	VIP Value	Identification
1	6.282	458.085	5.67	(−)—Epigallocatechin gallate
2	3.141	306.074	4.19	(−)—Epigallocatechin
3	4.659	290.079	3.82	(−)—Catechin
4	0.484	342.116	3.68	Coniferin
5	7.780	442.090	3.48	(−)—Epicatechin gallate
6	0.934	170.022	3.24	Gallic acid
7	6.283	480.067	3.07	Myricetin-3-*O*-galatoside
8	7.638	472.100	2.20	Methoxy-epigallocatechin gallate
9	7.780	464.072	2.05	Quercetin-3-*O*-glucopyranoside
10	6.282	916.168	2.04	Assamicain A/B/C
11	6.283	456.070	1.87	Methoxy-epicatechin gallate
12	0.501	174.100	1.85	*l*-theanine
13	0.497	534.179	1.75	Unknown
14	6.282	521.080	1.73	Unknown
15	0.483	388.122	1.66	Dihydrovomifoliol-*O*-glucopyranoside
16	0.493	516.216	1.64	Di-caffeoylquinic acid
17	0.484	405.112	1.64	Unknown
18	3.171	307.077	1.63	Unknown
19	0.551	406.109	1.53	Unknown
20	3.027	304.058	1.48	Taxifolin
21	7.160	458.085	1.45	(−)—Gallocatechin gallate
22	7.637	494.083	1.43	Unknown
23	3.143	374.061	1.43	Unknown
24	1.051	344.074	1.42	Theogallin
25	0.934	126.032	1.41	Unknown
26	4.662	358.066	1.41	Junipetrioloside A
27	6.280	548.054	1.38	Unknown
28	8.664	456.106	1.28	(−)—Epicatechin gallate-3”-*O*-Me
32	0.481	182.079	1.21	Unknown
34	5.104	578.142	1.11	Procyanidin B2
35	6.284	938.15	1.09	Unknown
37	6.278	454.054	1.06	Unknown
38	1.044	366.056	1.05	Unknown
39	6.281	478.051	1.03	7-methoxy-kaempferol-glucopyranoside
41	7.790	772.206	1.01	Quercetin 3-*O*-glucopyranosyl-*O*-glucopyranosyl-*O*-rhamnoside

Note: The abbreviations in the table are *t_R_*: retention time; MW: molecular weight; VIP value: the value of the variable influence on projection. The critical compounds are listed in order of VIP value from greatest to least.

**Table 4:** Pearson correlation coefficients between chemical compositions and biological activities.

Marker Compounds	Pearson Correlation Coefficient				
	ABTS	DPPH	FRAP	α-Amylase	α-Glucosidase
Myricetin-3-*O*-galactoside	0.719 *	0.713 *	0.799 **	0.552	0.724 *
Methoxy-epigallocatechin gallate	0.819 **	0.764 **	0.829 **	0.600	0.687 *
Quercetin-3-*O*-glucopyranoside	0.756 **	0.720 *	0.785 **	0.561	0.750 **
Assamicain A/B/C	0.832 **	0.765 **	0.817 **	0.586	0.661 *
Methoxy-epicatechin gallate	0.768 **	0.734 *	0.789 **	0.595	0.738 **
l-theanine	0.807 **	0.743 **	0.829 **	0.555	0.648 *
Trihydroxyflavone-di-arabinopyranoside	0.781 **	0.701 *	0.776 **	0.602 *	0.717 *
521.080 (unknown compound)	0.828 **	0.763 **	0.791 **	0.615 *	0.658 *
Dihydrovomifoliol-*O*-glucopyranoside	0.702 *	0.645 *	0.729 *	0.580	0.781 **
Di-*O*-caffeoylquinic acid	0.823 **	0.746 **	0.815 **	0.573	0.667 *
405.111 (unknown compound)	0.760 **	0.680 *	0.761 **	0.593	0.741 **
307.077 (unknown compound)	0.886 **	0.841 **	0.863 **	0.701 *	0.512
406.108 (unknown compound)	0.793 **	0.721 *	0.791 **	0.705 *	0.543
Pentahydroxyflavanone	0.889 **	0.840 **	0.854 **	0.745 **	0.423
494.061 (unknown compound)	0.793 **	0.733 *	0.822 **	0.528	0.703 *
374.061 (unknown compound)	0.880 **	0.830 **	0.834 **	0.713 *	0.516
Theogallin	0.108	0.075	0.242	0.045	0.764 **
126.031 (unknown compound)	−0.469	−0.431	−0.522	−0.304	−0.939 **
Junipetrioloside A	0.862 **	0.795 **	0.854 **	0.616 *	0.486
548.054 (unknown compound)	0.612 *	0.658 *	0.702 *	0.640 *	0.679 *
(−)—epicatechin gallate-3”-*O*-Me	0.791 **	0.750 **	0.811 **	0.598	0.720 *
182.079 (unknown compound)	−0.325	−0.285	−0.379	−0.224	−0.965 **
Procyanidin B2	−0.704 *	−0.568	−0.554	−0.278	−0.453
938.150 (unknown compound)	0.826 **	0.765 **	0.824 **	0.574	0.670 *
454.053 (unknown compound)	0.750 **	0.740 **	0.783 **	0.637 *	0.708 *
366.056 (unknown compound)	0.447	0.534	0.552	0.393	0.806 **
7-methoxy-kaempferol-glucopyranoside	0.585	0.589	0.716 *	0.411	0.751 **
Quercetin 3-*O*-glucopyranosyl-*O*-glucopyranosyl-*O*-rhamnoside	0.772 **	0.711 *	0.812 **	0.554	0.637 *
Aqueous extracts	0.55	0.633 *	0.768 **	0.376	0.553
Total polyphenols	0.620 *	0.599	0.727 *	0.479	0.736 **
Total amino acids	0.592	0.566	0.717 *	0.269	0.684 *
Total flavonoids	0.683 *	0.638 *	0.751 **	0.462	0.718 *
Total soluble sugars	0.364	0.351	0.485	0.350	0.688 *
Gallic acid	−0.272	−0.382	−0.329	−0.267	−0.748 **
Caffeine	0.207	0.294	0.406	0.084	0.199
GC	0.504	0.524	0.679 *	0.204	0.753 **
EGC	0.541	0.588	0.684 *	0.447	0.689 *
C	0.354	0.314	0.524	0.274	0.653 *
EGCG	0.510	0.528	0.682 *	0.445	0.714 *
EC	0.448	0.46	0.639 *	0.477	0.684 *
GCG	0.624 *	0.684 *	0.754 **	0.382	0.626 *
ECG	0.531	0.53	0.661 *	0.454	0.709 *
CG	0.277	0.075	0.314	0.210	0.373
Total catechins	0.535	0.555	0.698 *	0.421	0.718 *

Note: In one-way ANOVA analysis, **p < 0.01; * *p* < 0.05.

## Data Availability

Not available.

## Samples Availability

Samples of the compounds Gallic acid, caffeine, theobromine, (+)—catechin, (−)—epicatechin, (−)—gallocatechin, (−)—epigallocatechin, (−)—gallocatechin gallate, (−)—epigallocatechin gallate, and (−)—epicatechin gallate are available from the authors.
